# An Inductive Sensing System for Optimizing Prosthetic Socket Fit

**DOI:** 10.3390/s26154723

**Published:** 2026-07-25

**Authors:** Federico Andrei, Kim Baeten, Federico Donadel, Arianna Menciassi, Linda Paternò

**Affiliations:** 1 The BioRobotics Institute, Sant’Anna School of Advanced Studies, 56127 Pisa, Italy; federico.andrei@santannapisa.it (F.A.); k.baeten@student.utwente.nl (K.B.); federico.donadel@santannapisa.it (F.D.); arianna.menciassi@santannapisa.it (A.M.); 2 Faculty of Science and Technology (TNW), University of Twente, 7522 NB Enschede, The Netherlands

**Keywords:** inductive sensing, in-socket sensing, prosthetic socket fit, transfemoral prosthesis, residual limb volume, LC resonator, magnetic composite

## Abstract

**Highlights:**

**What is the main finding?**

- This study demonstrated that an inductive sensing system comprising a 1 mm thick silicone composite target made of Ecoflex™ 00-50 with 70 wt% NdFeB microparticles and an LC resonator based on a custom-made sensing coil (outer diameter = 28 mm, capacitance = 181 pF) can accurately detect distance changes within a 0–7.00 mm range and compression strains of up to 50%. These results suggest that this approach could represent a promising solution for monitoring prosthetic socket fit in transfemoral users.

**What is the implications of the main finding?**

- The developed system can be integrated into a transfemoral prosthetic socket to continuously monitor socket fit, which may be affected by residual limb volume fluctuations over time. These data could provide objective information on socket-fit changes and may support future socket-adjustment decisions after validation in prosthesis users.

**Abstract:**

This work presents the design, development, and experimental validation of an inductive sensing system for monitoring prosthetic socket fit variations caused by residual limb volume fluctuations. The system aims to reduce the risk of discomfort and tissue injury by measuring the distance between the outer rigid socket and the inner silicone elastomeric liner worn in direct contact with the residual limb. The sensing architecture consists of a portable data acquisition unit, an LC resonator sensor mounted on the inner surface of the rigid socket, and a magnetic silicone target attached to the external surface of the liner. Multiple configurations of LC resonators and magnetic targets were designed and evaluated. The results indicate that a medium-sized coil (outer diameter = 28 mm, capacitance = 181 pF) combined with a 1 mm thick silicone target made of Ecoflex™ 00-50 with 70 wt% NdFeB microparticles provides the most stable and sensitive performance. Experiments demonstrated stable distance detection up to 7 mm, with the resonant-frequency shift (relative to the baseline condition) varying from −31.37 kHz at 0 mm to −2.94 kHz at 7.00 mm, for a total shift range of 29.84 kHz. Environmental tests showed minimal drift, with frequency variations below 0.40 kHz across temperature (25–60 °C) and humidity (50–90% RH) changes. In vitro validation using a high-fidelity residual limb simulator and an adjustable socket reproduced controlled residual limb volume variations of 300 mL (i.e., +7.5%), resulting in repeatable resonant-frequency changes within 3.15–3.17 MHz with measurement variability ($u_{A}$, Type A) below 0.13 kHz.

## 1. Introduction

Limb amputation represents a major global health challenge, as it is associated with profound physical, psychological, and social impairments that substantially affect quality of life. In 2021, an estimated 445.2 million individuals worldwide were living with traumatic limb amputation, with approximately 10 million new cases occurring in that year, according to a recent Global Burden of Disease analysis [1]. Beyond traumatic causes, the overall burden of limb amputation is further amplified by non-traumatic etiologies, particularly the rising incidence of diabetes and peripheral vascular diseases, which account for a substantial proportion of amputations in many regions worldwide [2,3,4]. Of these patients, around 90% have lower-limb amputations, and among lower-limb cases, approximately 26% correspond to transfemoral amputations [5,6]. In this already critical context, the global incidence and prevalence of limb amputation are expected to rise further due to the increasing prevalence of diabetes and peripheral vascular disease, population aging, and conflict-related traumatic injuries [2,5,6].

Within this scenario of increasing clinical burden and long-term disability, effective rehabilitation strategies become essential. Prosthetic devices play a fundamental role in enabling functional recovery, social reintegration, and overall improvement in quality of life for individuals with limb amputation. However, the effectiveness of prosthetic rehabilitation largely depends on the quality of the biomechanical coupling between the residual limb and the prosthetic device. In particular, for people with lower-limb amputation, both clinicians and users consistently identify socket-related issues as a primary determinant of prosthesis comfort, function, and long-term use [7,8]. As the socket represents the physical interface between the residual limb and the prosthesis, achieving and maintaining optimal socket fit is paramount to maximizing mobility and preserving residual limb tissue health, thereby supporting successful rehabilitation.

The challenge of achieving and maintaining optimal socket fit is largely driven by fluctuations in residual limb volume over time, which arise from multiple factors and vary considerably among individuals [9]. These volume fluctuations can lead to poor prosthetic socket fit and significantly impact daily prosthesis use and long-term comfort. For example, a recent investigation in individuals with transfemoral amputation demonstrated that residual limb volume increases significantly following prosthesis removal and physical activity [10]. Measured post-doffing volume changes were shown to follow a two-term exponential decay, with the largest increase occurring within the first 10 min and an average stabilization time of approximately 30 min. Across individuals, peak post-doffing volume increases of up to about 6% were observed. In more severe cases, these volume variations may hinder prosthesis donning [9,10]. Additionally, such volume variations alter the distribution of pressures at the tissue–socket interface, where elevated mechanical loads are already typically observed during daily activities. Quantitative measurements have reported interface pressures ranging from approximately 40 kPa during swing phases to over 110 kPa during stance, depending on the locomotion task and socket region [11]. As a consequence, poor socket fit can result in skin breakdown, pressure sores, musculoskeletal overuse injuries, and osteoarthritis [12].

Accordingly, it is essential for individuals with limb amputation to be able to recognize when socket fit requires adjustment in order to enable timely intervention. However, such changes are often difficult for users to perceive in their early stages. When socket-related issues become evident, individuals typically need to attend a prosthetic clinic, where socket modifications involve time-consuming rectification procedures. These delays can result in prolonged exposure to suboptimal fit conditions, leading to discomfort, skin irritation, and other adverse symptoms that may compromise prosthesis use and rehabilitation outcomes [10]. For this reason, there is a clear need for a system capable of objectively detecting when socket-fit adjustment is required, thereby enabling timely interventions and preventing the onset of adverse symptoms.

Several measurement systems have been developed to monitor prosthetic socket fit, with many approaches focusing on measuring pressures at the limb–socket interface as an indirect indicator of fit quality [13]. Among these, force-sensitive resistors have been widely employed due to their low cost, thin profile, ease of integration, and minimal impact on socket fit [14,15]. However, their application is limited by poor repeatability, pronounced signal drift under static and cyclic loading, and sensitivity to loading history, surface curvature, and combined compression-shear conditions typical of the prosthetic socket environment. In addition, force-sensitive resistors’ performance is affected by environmental factors such as temperature and humidity, resulting in reduced measurement accuracy and reliability for long-term monitoring. Fluid-filled pressure sensors transmit applied loads through a confined fluid to a pressure transducer, rather than directly sensing force at the contact interface. A MEMS-based bubble pressure sensor has been proposed for prosthetic socket interface pressure measurement, demonstrating excellent drift performance and good pressure resolution, with some hysteresis attributed to the surrounding silicone material [16]. Despite these advantages, fluid-filled sensor designs remain largely experimental: the requirement for fluid containment introduces potential concerns related to leakage and long-term robustness [17], while reviews of pressure measurement systems for prosthetic sockets identify sensor thickness, packaging complexity, and the potential increase in system weight when multiple sensing elements are employed as key limitations for integration within liners or sockets [18]. Fiber optic sensors, particularly fiber Bragg grating-based systems, offer high sensitivity, excellent linearity, negligible signal drift, and immunity to electromagnetic interference, making them attractive for measuring pressures at the limb–socket interface [19,20]. However, despite these advantages, fiber-optic sensing solutions are typically fragile, require careful embedding and protection, and rely on complex, bulky, and costly interrogation units. As a result, their use has so far been largely confined to laboratory settings, limiting their widespread adoption in routine clinical practice. Capacitive pressure sensors represent another promising alternative and have demonstrated high sensitivity, good accuracy, reduced hysteresis and drift, and lower temperature dependence compared to resistive technologies [14,21]. Recent developments include elastomeric, textile-based, and flexible capacitive sensor arrays that can be integrated into liners or placed at the limb–socket interface to provide distributed pressure measurements. Despite these advantages, many capacitive systems require complex electronics and careful shielding to mitigate crosstalk and electromagnetic interference. An additional major challenge associated with sensors that measure pressures at the socket–residual limb interface, regardless of the sensing technology, arises from the fact that interface pressures vary continuously throughout the gait cycle, following a characteristic double-peaked (M-shaped) pattern, with low values during swing and two distinct maxima during stance [22,23]. Quantitative analyses in people with transfemoral amputation have shown that interface pressures can fluctuate by tens of kPa within a single step [11]. Consequently, it becomes difficult to discriminate whether observed pressure changes are caused by physiological variations in residual limb volume, which would require socket adjustment, or are merely the result of normal gait dynamics. For this reason, several approaches have highlighted the importance of assessing socket fit under quasi-static conditions, such as double-support standing, where pressure distributions are more stable and user-specific [24,25].

An alternative strategy is to measure the distance between the prosthetic socket and the liner, which provides a more direct assessment of socket looseness and overall fitting quality [26]. Since the liner is a compliant, closely fitting silicone sock worn directly on the residual limb, variations in liner–socket distance largely reflect changes in the socket–residual limb distance. These distance variations are primarily driven by changes in residual limb volume and are less affected by the dynamic loading patterns associated with gait. In this regard, inductive sensing is a particularly promising technology for this application due to its high resolution, stability over time, and low sensitivity to temperature, humidity, and loading history [26]. Inductive sensing relies on an LC resonant circuit, in which the resonant frequency depends on the inductance of a sensing coil. The presence and proximity of a magnetic silicone target alter the magnetic coupling with the sensing coil, modifying its effective inductance and, consequently, shifting the resonant frequency of the LC circuit. When adapted to prosthetic applications, the sensing coil can be embedded within the socket wall, while the corresponding magnetic target is integrated into the liner. Variations in the distance between these two components result in measurable frequency shifts, enabling continuous monitoring of liner–socket distance changes that are directly associated with socket fit [27].

To address these limitations, the present study reports a systematic cross-comparison of multiple coil–target configurations combined with magnetic targets containing different NdFeB particle concentrations and thicknesses. The system also introduces direct resonant-frequency monitoring and a portable wireless electronics unit based on an Arduino Nano 33 BLE, enabling continuous and practical use outside controlled laboratory environments. Reliability was further examined through environmental characterization in a climatic chamber under varying temperature and humidity conditions. Key performance metrics, including frequency sensitivity, signal stability, noise level, quality factor, and maximum detectable distance were used to identify the optimal sensor–target combination. Finally, the selected configuration was integrated into a transfemoral prosthetic socket and liner and experimentally validated using a variable-volume transfemoral residual limb simulator. This experimental setup enabled controlled and repeatable validation of the system’s ability to detect volume-induced fitting changes under realistic conditions, providing stronger evidence for future clinical translation.

## 2. Materials and Methods

### 2.1. System Overview

**Figure 1:**
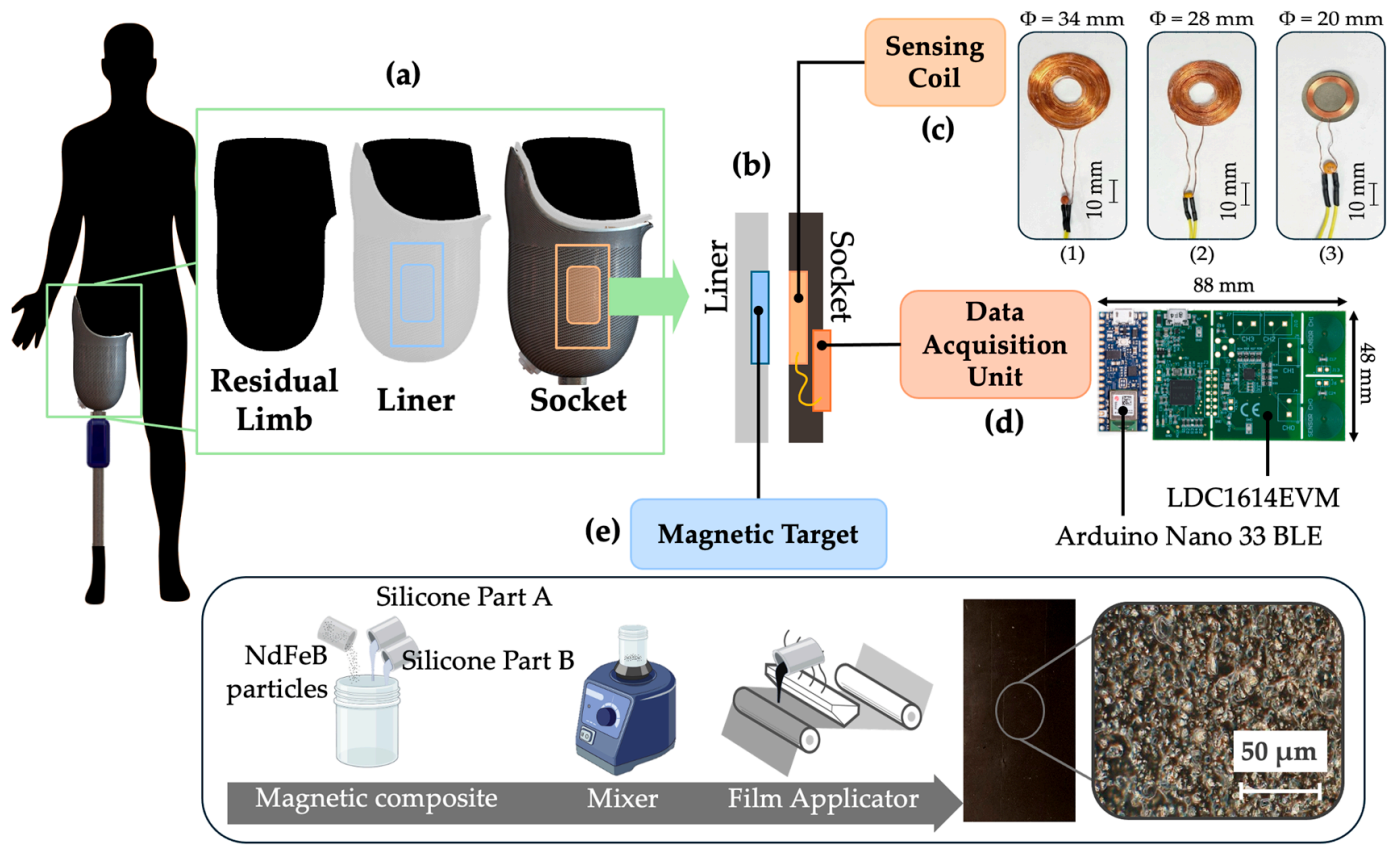
Overview of the inductive sensing system integrated into a transfemoral prosthesis. (**a**) Schematic representation of the prosthetic configuration, showing the residual limb, elastomeric liner, and rigid socket. The highlighted regions indicate the sensing area within the socket–liner interface. (**b**) Cross-sectional diagram of the sensing architecture and detection principle. A single planar inductive coil embedded in the socket wall forms the LC resonator, while a magnetic silicone layer integrated into the liner acts as the magnetic target. Variations in the coil–target distance modulate the resonant frequency, which is measured by the data acquisition unit. On the external surface of the socket are (**c**) planar inductive coils, evaluated during the design optimization phase: custom coils, with diameters of 34 mm (1) and 28 mm (2), and an industrial 20 mm coil (3). (**d**) The selected coil interfaces with an LDC1614EVM and an Arduino Nano 33 BLE microcontroller. (**e**) The fabrication process of the magnetic targets. NdFeB microparticles were dispersed into a two-component silicone elastomer (Ecoflex™ 00-50), homogenized, and cast into thin films to obtain the magnetic target. The optical micrograph shows the distribution of NdFeB particles within the silicone matrix and was acquired using a digital optical microscope (HRX-01 3D Digital Microscope, Hirox Co., Ltd., Tokyo, Japan) equipped with a wide-range objective lens at a magnification of 700×.

The developed inductive sensing system comprises the following three main components: a portable data acquisition unit, an LC resonator sensor, and a magnetic target, as illustrated in Figure 1. The data acquisition unit consists of an LDC1614 Evaluation Module (Texas Instruments, Dallas, TX, USA), which is connected to an Arduino Nano 33 BLE microcontroller (Arduino S.r.l., Monza, Italy). The LC resonator comprises a planar inductive sensing coil connected to a capacitor, while the sensing target consists of a silicone layer containing NdFeB magnetic microparticles. The following sections describe each component in detail.

#### 2.1.1. Data Acquisition Unit

The LDC1614EVM Evaluation Module uses inductive sensing technology to sense the proximity of a magnetic target. An inductance-to-digital converter (LDC) provides an alternating current to drive the LC resonator, matching its resonant frequency. The resonant frequency changes when the LC resonator is in close proximity to a magnetic target due to a change in the self-inductance or mutual inductance of the inductor. This change is calculated using Equation (1) [28]:

(1) $$f_{sensor}=\frac{1}{2\pi \sqrt{LC}}$$

where L represents the inductance (H), and C represents the capacitance (F). An Arduino Nano 33 BLE microcontroller with integrated Bluetooth^®^ Low Energy (BLE) connectivity was used to enable a fully portable data acquisition unit. The LDC1614EVM and the Arduino Nano 33 BLE were connected via an I2C interface. The Arduino Nano 33 BLE was programmed using the Arduino IDE (version 2.3.6) following the register map, configuration parameters, and computational instructions provided in the official Texas Instruments LDC1614 datasheet. The LDC1614 was configured for continuous acquisition. After each acquisition cycle, the Arduino transmitted the resonant-frequency data to MATLAB_R2026a through the serial interface. Although the firmware included a programmed delay of 50 ms after each acquisition cycle, the effective sampling rate, determined from the recorded timestamps, was approximately 17 Hz across all experiments. The first 100 samples of each recording were discarded to remove acquisition transients. For transition detection, the signal was smoothed using a 20-sample moving-minimum filter followed by a 20-sample moving-average filter. Step transitions were automatically identified using derivative-based adaptive thresholding.

#### 2.1.2. LC Resonator Sensor

The LC resonator consists of a sensing coil connected in parallel to a capacitor, forming a parallel LC tank circuit. Three LC resonator configurations were tested, as follows: one incorporating an industrial coil and two based on custom-fabricated coils, as shown in Figure 1c. The custom-fabricated coils were manufactured by winding copper wire (diameter: 0.30 mm/AWG 29; Mouser Electronics, Mansfield, TX, USA) onto a plexiglass support. The wire turns were secured using a cyanoacrylate adhesive (Loctite Super Attak^®^, Henkel, Italy). The industrial coil consisted of a miniature receiving coil manufactured by TDK Corporation, Tokyo, Japan (product number: WR202010-18M8-ID). After being connected in parallel to capacitors, the resulting LC resonators were characterized in terms of resonant frequency, quality factor, and measured electrical parameters, including resistance and inductance. The resonant frequency was obtained from complex-impedance measurements performed using a DG8SAQ Vector Network Analyzer v3 (VNA; SDR-Kits, Kent, UK). The inductance and quality factor values were derived from the mean impedance response of the resonators measured with the VNA. The electrical resistance was measured using a handheld LCR meter (MS8217, Mastech Group, Shenzhen, China). Among the tested configurations, the medium LC resonator showed the highest quality factor (Q = 21.7). All measurements were repeated three times, and the values reported in Table 1 correspond to the mean ± standard deviation. The geometric parameters of the inductive sensing coils are also summarized in Table 1.

#### 2.1.3. Magnetic Target

Five different magnetic targets were produced, varying in magnetic particle concentration and thickness. Specifically, the targets were fabricated by first weighing the two components of the silicone elastomer Ecoflex™ 00-50 Platinum Silicone Rubber (Smooth-On, Macungie, PA, USA). Neodymium–iron–boron (NdFeB) hard magnetic microparticles (average size: 5 μm; MQP-15-7-20065, Magnequench, Germany) were then added to the silicone matrix and mechanically mixed (Simply Mixer, IGT Testing Systems, Amsterdam, The Netherlands) for 15 s to ensure homogeneous dispersion. In particular, samples were prepared with two particle concentrations, 60 wt% and 70 wt%. Subsequently, the prepared magnetic silicone mixtures were cast using a mechanical film applicator (Zehntner GmbH Testing Instruments, Sissach, Switzerland) set to two target thicknesses, 0.5 mm and 1.0 mm. All samples were cured for 4 h at room temperature. Then, the actual thickness of each magnetic layer was measured using an Outside Micrometer caliper (0–25 mm ± 0.01, RS components, Milano, Italy), and the resulting values are reported in Table 2. Although a calibrated film applicator was used, the final thicknesses slightly differed from the nominal settings. These deviations can be attributed to factors such as the high particle loading, the non-Newtonian behavior of the composite mixture, minor material shrinkage during curing, and edge effects during film casting. The magnetic targets had a diameter of 100 mm, substantially larger than the sensing coils, in order to minimize edge effects and provide approximately constant boundary conditions during the experiments. Consequently, the characterization focused on variations in the coil–target separation, while the influence of lateral coil–target misalignment was intentionally minimized.

### 2.2. Experimental Setting

#### 2.2.1. Distance–Frequency Characterization

**Figure 2:**
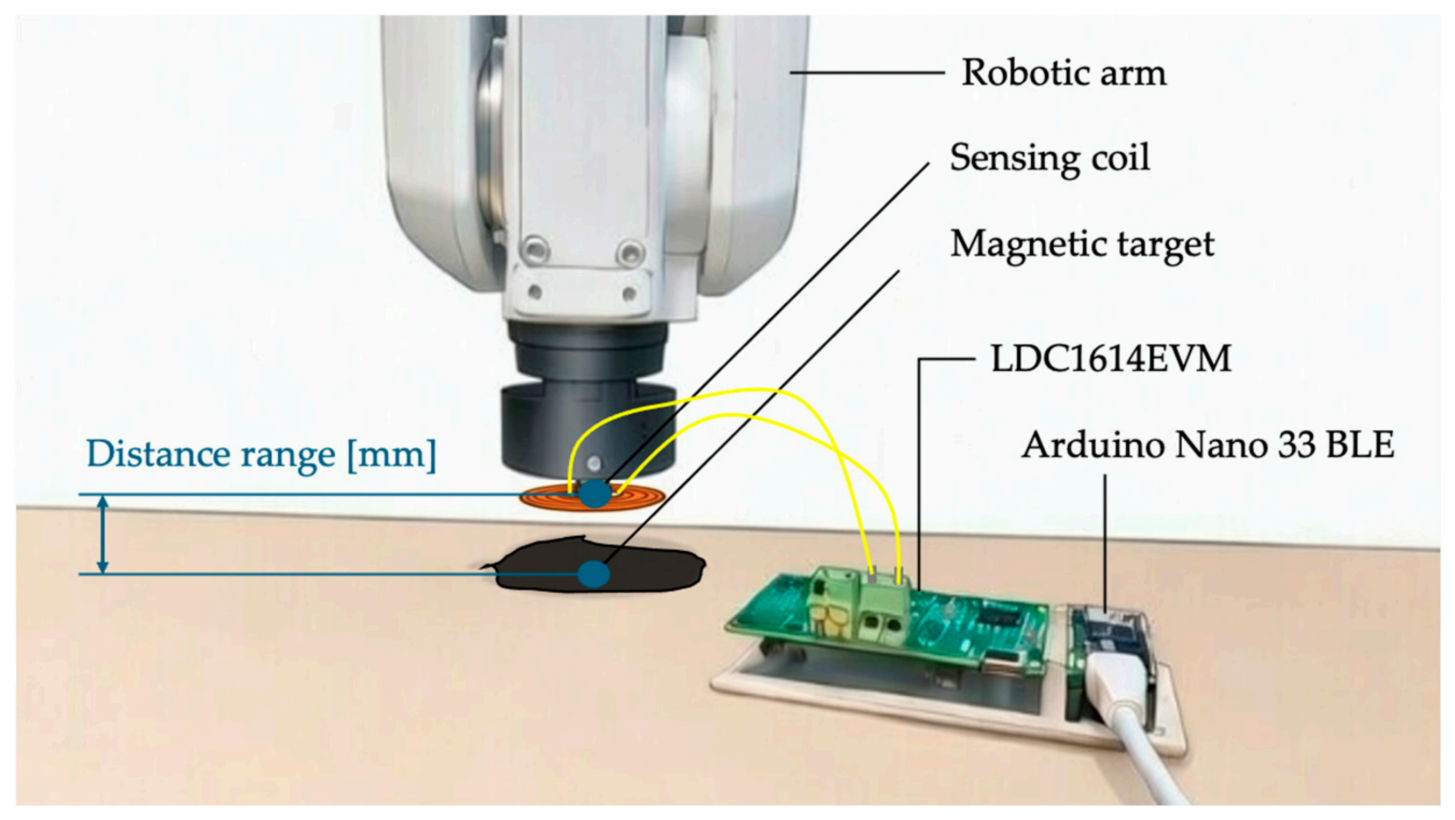
Experimental setup for the distance–frequency characterization. A robotic arm controls the vertical displacement of the sensing coil over a 0–10 mm range relative to the magnetic target. The sensing coil is mounted on the arm end-effector, while the magnetic target is fixed on a flat surface below. Data acquisition is performed via the LDC1614EVM connected to the Arduino Nano 33 BLE.

The resonant-frequency variation was characterized over a 10 mm distance range. The sensing coil was fixed to a robotic arm end-effector, while the magnetic target was positioned on a flat support beneath the arm, as shown in Figure 2. At the beginning of each measurement sequence, the coil was positioned at a distance of 10 mm from the magnetic target (baseline). The robotic arm then moved the coil progressively downward until a separation of 0 mm was reached. After contact, the distance was increased again in increments of 1 mm, following the same positions used during the descent. At each distance step, approximately 100–200 resonant-frequency samples were acquired during the short-duration tests, corresponding to an acquisition time of approximately 10 s at the effective sampling rate of 17 Hz (short-duration tests). This complete sequence was repeated three times for every coil–target combination.

These tests followed a progressive selection strategy. Initially, all five magnetic targets (varying in particle concentration and thickness, Table 2) were tested with the industrial LC resonator (Test 1) and subsequently with the medium custom-fabricated LC resonator (Test 2). This systematic comparison enabled evaluation of the influence of target thickness and particle concentration on frequency sensitivity, signal stability, noise level, and measurable distance range under identical operating conditions. From these tests, the 1.00 mm thick magnetic target (70 wt% NdFeB) consistently provided the largest resonant-frequency variation and the most stable response. Based on these findings, subsequent tests involving the large LC resonator were conducted exclusively with the 1.00 mm thick magnetic target (Test 3), thereby avoiding redundant measurements with configurations that had already shown inferior performance in previous tests (with both the industrial and medium LC resonators). This additional test was performed to assess whether increasing the coil dimensions could further improve sensing performance. Among all these tested combinations, the medium LC resonator paired with the 1 mm target demonstrated the most stable and low-noise response, together with the largest measurable displacement range. This configuration was, therefore, selected as the optimal sensing setup and used for longer-duration distance tests (Test 4). These tests followed the same distance trajectory as described above (drop of 10–0 mm and step-up of 0–10 mm at a 1 mm step), while maintaining the same effective sampling rate (17 Hz). The acquisition period was extended to approximately 50–60 s per step, resulting in 800–1000 samples per step. The detectable distance range was defined as the largest continuous interval satisfying |Δf(d)|≥2  SD(d), where SD(d) is the local standard deviation calculated from the repeated measurements at each distance. Because the sensor response is non-linear, the local sensitivity was evaluated at representative distance intervals using the finite-difference approximation $S(d_{i})=({∆f}_{i+1}-{∆f}_{i})/(d_{i+1}-d_{i})$ and reported both near the contact region (0–1 mm) and near the detection limit. Finally, the corresponding local distance resolution was estimated to be ∂d=2  SD(d)/S(d).

Table 3 summarizes all of the tested LC resonator–target combinations and experimental conditions. All measurements were repeated three times (N = 3). After automatic detection of the transition points, the resonant frequency corresponding to each distance step was estimated to be the arithmetic mean of the processed resonant-frequency samples acquired during the steady-state plateau between two consecutive transitions. The baseline frequency ($f_{baseline}$) was defined as the mean resonant frequency measured during the initial 10 mm plateau, before the first displacement step toward the target was applied. The resonant-frequency shift was then computed as Δ*f*(*d*) = *f_measured_*(*d*) − *f_baseline_*, where *f_measured_*(*d*) denotes the mean resonant frequency at distance d. Therefore, the reported frequency shifts represent changes relative to the baseline condition rather than differences between consecutive distance steps. Consequently, the baseline condition (10 mm) corresponds to Δ*f* = 0. Measurement uncertainty is reported as the Type A standard uncertainty of the mean, calculated as $u_{A}=SD/\sqrt{N}$ (k=1), where SD is the sample standard deviation of the repeated measurements. Because of the limited number of repetitions, the corresponding two-sided 95% confidence interval of the mean was estimated to be $CI95=t_{0.975,2}u_{A}$, where $t_{0.975,\nu }$ is the Student’s t-factor for a two-sided 95% confidence interval (ν=N−1=2). The total resonant-frequency shift range was defined as the absolute difference between the frequency shifts measured at 0 mm and maximum tested sensor–target separation. The detectable distance range was defined as the largest continuous interval satisfying |Δf(d)|≥2  SD(d), where SD(d) is the local standard deviation calculated from the repeated measurements at each distance. Because the sensor response is non-linear, the local sensitivity was evaluated at representative distance intervals using the finite-difference approximation $S(d_{i})=({∆f}_{i+1}-{∆f}_{i})/(d_{i+1}-d_{i})$ and reported both near the contact region (0–1 mm) and near the detection limit. Finally, the corresponding local distance resolution was estimated to be ∂d=2  SD(d)/S(d).

#### 2.2.2. Compression–Frequency Characterization

Compression tests were conducted to determine whether deformation of the magnetic silicone target produced measurable variations in the resonant frequency. Based on the results obtained in the distance–frequency characterization, only the medium LC resonator combined with the 1 mm thick magnetic target was used for these tests, as this configuration had demonstrated the highest sensitivity and signal stability. These experiments were performed using the same robotic arm setup employed for the distance–frequency characterization (Figure 2). Prior to the compression tests, the robotic arm was calibrated with respect to the reference table surface. The nominal 0% compression condition was defined from the known undeformed thickness of the 1 mm magnetic target, corresponding to the onset of contact between the target and the sensing coil, and was verified by visual inspection. Compression levels of 25% and 50% were then achieved by applying additional programmed downward displacements corresponding to 25% and 50% of the target thickness, respectively.

At the beginning of each trial, the coil was positioned at a distance of 10 mm from the magnetic target (baseline). The robotic arm then applied a controlled downward displacement to compress the target to 50% of its original thickness, and resonant-frequency data were recorded for approximately 12 s. After this first measurement, the arm was retracted to achieve a 25% compression level, which was again recorded for approximately 12 s. The load was then fully released, returning the target to 0% compression, and a final measurement was acquired. This entire sequence (baseline-50–25–0% compression) was executed three times (Test 5).

Longer-duration compression tests (Test 6) followed the same compression sequence and maintained the same effective sampling rate (17 Hz). The acquisition period was extended to approximately 60 s per compression level, yielding approximately 1000 samples for each plateau.

#### 2.2.3. Temperature and Humidity Stability Tests

Environmental stability tests were performed to evaluate the influence of temperature and humidity on the resonant frequency of the inductive sensing system, thereby providing insight into the potential effects of elevated in-socket temperatures and perspiration during daily prosthesis use [29]. The medium LC resonator and the 1 mm magnetic silicone target were mounted together inside a Temperature and Humidity Chamber (Model SH-242, ESPEC Corporation, Osaka, Japan). A small access port on the chamber wall allowed routing of the sensor wiring to the external data acquisition unit (LDC1614EVM and Arduino Nano 33 BLE), ensuring stable electronic operation outside of the controlled environment while exposing only the sensing components to the temperature and humidity conditions.

Two separate experiments were carried out. In the first test, the aim was to evaluate system stability under varying temperature (Test 7). The relative humidity inside the chamber was held constant at 50%, while the temperature was gradually increased from 25 °C to 60 °C in a uniform and continuous manner. Throughout the temperature sweep, the coil and target remained at a fixed separation of 0 mm. Resonant-frequency data were collected continuously, and the full experiment was repeated three times to ensure repeatability. A second experiment was then performed to assess the sensitivity of the system to humidity changes (Test 8). In this case, the chamber temperature was kept constant at 25 °C, while the relative humidity was gradually increased from 50% to 90%, again following a uniform progression. As in the temperature test, the sensor components were maintained at 0 mm separation, and three repeated trials were conducted to quantify variability.

### 2.3. In Vitro Validation

In vitro validation tests were conducted to assess the performance of the sensing system under controlled conditions using an adjustable transfemoral prosthetic socket and a high-fidelity residual limb simulator, see Video S1 in the Supplementary Materials. Specifically, the experiments were performed with a 3D-printed volume-adjustable transfemoral socket previously described by Donadel et al. [30]. The socket was fabricated primarily from electrically non-conductive polymer-based materials (glass-filled PA12) and, therefore, did not interfere with the magnetic coupling between the LC resonator and the magnetic target or influence the resonant-frequency measurements. This socket integrates a manual adjustment mechanism based on a BOA^®^ closure system that controls a cable-driven structure. By tightening or loosening the BOA dial, the cable system modifies the circumferential constraint of the socket, thereby increasing or decreasing its internal volume in a controlled and repeatable manner. The socket is coupled with a silicone liner through a magnetic suspension system. For the present study, three distinct socket-width configurations were manually set using the BOA mechanism to reproduce different levels of socket tightness while maintaining continuous liner–socket contact. Controlled inflation-deflation cycles of the residual limb simulator were then performed to induce residual limb volume fluctuations, thereby generating variations in interface pressure and magnetic-target compression within each configuration. A high-fidelity transfemoral residual limb simulator, described by Paternò et al. [24], was used to reproduce realistic soft-tissue interaction. The simulator integrates an internal fluidic chamber connected to two syringe pumps (BS-8000; Braintree Scientific Inc., Braintree, MA, USA), allowing for precise modulation of limb volume through controlled fluid injection and withdrawal. For each socket configuration, the simulator volume was increased and decreased by 7.5% by injecting or removing 300 mL of water per cycle at a constant rate of 50 mL/min, and three complete inflation–deflation cycles were performed to ensure repeatability. The 1 mm thick magnetic target was bonded to the external surface of the silicone liner worn on the simulator using silicone adhesive (Sil-Poxy^®^, Smooth-On, Macungie, PA, USA). The medium LC resonator, previously identified as the optimal sensing configuration, was integrated into the internal surface of the prosthetic socket at the corresponding sensing location. The portable data acquisition unit was mounted on the outer socket surface and used to continuously record the resonant frequency, which reflects changes in the coil–target coupling induced by variations in fitting conditions and target compression, during both inflation and deflation phases of the simulator across all three socket configurations. Hysteresis was quantified by comparing the inflation and deflation branches after mirroring the inflation branch with respect to the turning point of the cycle. The average absolute frequency difference was then calculated over ten corresponding points (uniformly spaced between the turning point and the end of the deflation) around each displacement level. This experimental setup enabled evaluation of the system’s capability to detect socket–liner distance variations induced by controlled residual limb volume changes under different fitting conditions.

## 3. Results

### 3.1. Distance–Frequency Characterization

**Figure 3:**
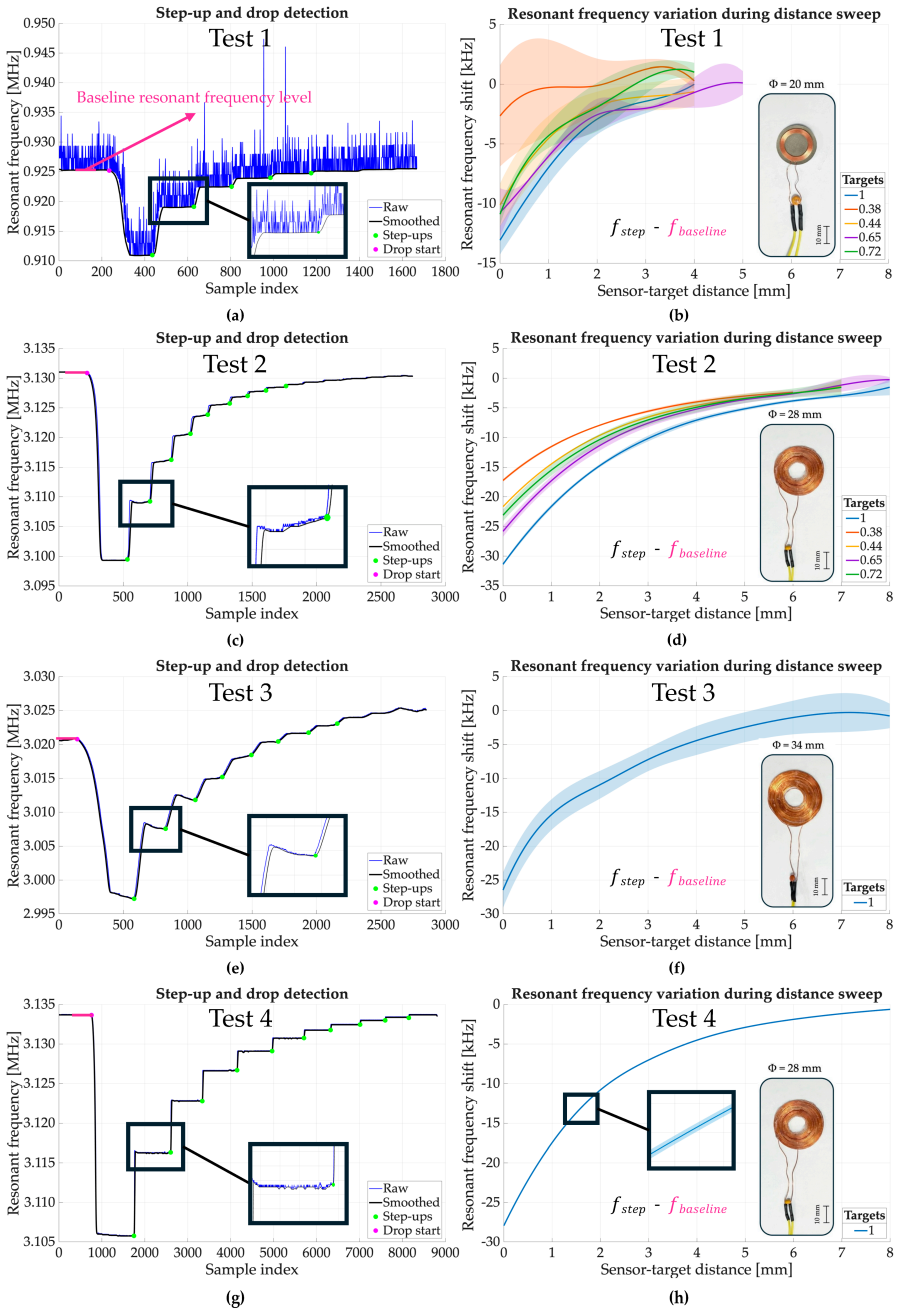
Resonant-frequency measurements obtained during the distance–frequency characterization for the industrial, medium, and large LC resonators. Industrial LC resonator: (**a**) measured resonant-frequency signal for the 1.00 mm target during the full sweep cycle (10–0 mm drop followed by 1 mm step-up increments), including the smoothed trace and automatically detected transition points. (**b**) Corresponding resonant-frequency shifts over the 0–8.00 mm range for all magnetic target thicknesses, computed relative to the pre-drop baseline. Medium LC resonator: (**c**) measured resonant-frequency signal for the 1.00 mm target during the same sweep cycle, with smoothed trace and transition-point detection. (**d**) Resonant-frequency shifts over 0–8.00 mm for all targets, referenced to the baseline. Large LC resonator: (**e**) measured resonant-frequency signal for the 1.00 mm target during the same sweep cycle, with smoothed trace and transition-point detection. (**f**) Corresponding resonant-frequency shift over 0–8.00 mm, relative to the baseline. Medium LC resonator (long-duration test): (**g**) resonant-frequency recording for the 1.00 mm target during long-duration acquisition, including the sweep cycle and transition-point detection. (**h**) Corresponding resonant-frequency shift over 0–8.00 mm, referenced to the baseline. Shaded regions indicate the standard deviation. For all right-hand panels, the continuous curves were obtained by cubic-spline interpolation of the discrete experimental measurements.

The test with the industrial LC resonator shows a stable but noisy signal, as depicted in Figure 3a. Data analysis shows that this LC resonator has a sensing distance of 2.00 mm for the 1.00 mm thick target, 1.00 mm for the 0.72 mm, 0.65 mm, and 0.44 mm thick targets, and below 1.00 mm for the 0.38 mm thick target, as shown in Figure 3b and summarized in Table 4. Beyond these thresholds, the resonant-frequency variation becomes comparable to, or falls below, twice the local standard deviation associated with the resonant-frequency shift measurements, thus preventing reliable discrimination of further changes in target–sensor separation (|Δf(d)|≥2 SD(d)). Table 4 summarizes the results obtained with the industrial LC resonator for all five magnetic target configurations. For each target, the table reports the frequency-shift of the mean curve relative to the baseline value (defined as the resonant frequency measured at a 10 mm sensor–target separation) at 0 mm and at the maximum detectable distance (mm), the corresponding total frequency shift, the mean standard deviation of the resonant-frequency measurements (SD), the Type A standard uncertainty of the mean ($u_{A}$), the corresponding 95% confidence interval, the detectable distance range, and the local sensitivity with the estimated distance resolution at representative operating points. Among the tested targets, the 1.00 mm thick target demonstrated the largest frequency variation range (13.07 kHz) combined with a comparatively low standard uncertainty of the mean (0.59 kHz). Nevertheless, the 0.65 mm target also exhibited a notably stable response, showing the lowest standard uncertainty of the mean among all tested specimens (0.57 kHz) while maintaining a substantial overall frequency range (10.87 kHz).

The test with the medium LC resonator shows a stable and less noisy signal, as shown in Figure 3c. Analysis of the data in Figure 3d and Table 5 shows that this LC resonator has a sensing distance of 7.00 mm for the 1.00 mm thick target and 6.00 mm for the 0.72 mm, 0.65 mm, 0.44 mm, and 0.38 mm thick targets. Table 5 summarizes the resonant-frequency shift ranges (relative to the baseline value) together with the corresponding measurement variability (Mean SD), the Type A standard uncertainty ($u_{A}$), the corresponding 95% confidence interval, the detectable distance range, and the local sensitivity and estimated distance resolution at representative operating points, obtained with the medium LC resonator for all five magnetic target configurations. As observed with the industrial coil, the 1.00 mm thick target provided the largest frequency variation range (29.84 kHz) while maintaining a relatively small standard uncertainty of the mean (0.25 kHz), confirming its robust performance under both resonator configurations.

The test with the large coil LC resonator shows an unstable signal, as seen in Figure 3e. During the fixed-distance plateaus, the resonant frequency exhibited a noticeable drift rather than remaining constant. Consequently, the same resonant-frequency values could correspond to multiple sensor–target distances, compromising the uniqueness of the frequency-based distance encoding and reducing the reliability of the distance estimation. Accordingly, the standard uncertainty of the measurements was higher compared to those obtained with the medium and industrial LC resonators. Measurements of the resonant-frequency shift as the sensor–target distance varied yielded a standard uncertainty of the mean ($u_{A},\;$Type A) of 1.29 kHz and a 95% confidence interval (CI) of ±5.55 kHz for the 1.00 mm thick target. The large LC resonator demonstrated a detectable distance range of up to 4.00 mm for the 1.00 mm thick target, according to the same local 2SD(d) criterion adopted for the previous resonators, as illustrated in Figure 3f. The corresponding total resonant-frequency shift range of the mean curve was 26.16 kHz (with a resonant-frequency shift of −26.47 kHz at 0 mm and –4.43 kHz at the detection limit). The local sensitivity decreased from 11.10 kHz/mm near the contact point (0–1.00 mm) to 2.80 kHz/mm near the detection limit (3.00–4.00 mm), while the corresponding estimated distance resolution degraded from 0.47 mm to 1.22 mm. Beyond 4 mm, the resonant-frequency shift became comparable to the local measurement variability, preventing reliable discrimination of additional changes in sensor–target separation.

A comparison of the 0–10 mm distance test results obtained with the industrial, medium, and large LC resonators using the 1.00 mm thick target (Table 6) indicates that the medium LC resonator provides the best overall performance. Specifically, it exhibits lower noise than the industrial coil and greater signal stability than the large coil. Additionally, it has a sensing range of 7.00 mm for the 1.00 mm thick target, which is larger than the sensing range of the large and industrial coils. It also shows the greatest resonant frequency shift range in the signal, equal to 29.84 kHz, compared to 13.07 kHz for the industrial coil and 26.16 kHz for the large coil.

In the longer-duration test, the medium LC resonator paired with the 1 mm thick target maintained a stable signal, as depicted in Figure 3g, consistent with the previous short-duration results. Measurements of the resonant frequency shift as the sensor–target distance varied yielded a standard uncertainty of the mean ($u_{A},\;$Type A) of 0.03 kHz and a 95% confidence interval (CI) of ± 0.15 kHz. The maximum measurable distance range was 8.00 mm, according to the same local 2SD(d) criterion adopted for the previous resonators. The corresponding total resonant-frequency shift range of the mean curve was 27.77 kHz (with a resonant-frequency shift of −27.92 kHz at 0 mm and –0.62 kHz at the detection limit), as shown in Figure 3h. The local sensitivity decreased from 10.46 kHz/mm near the contact point (0–1.00 mm) to 0.54 kHz/mm near the detection limit (7.00–8.00 mm), while the corresponding estimated distance resolution increased from 0.01 mm to 0.02 mm.

### 3.2. Compression–Frequency Characterization

**Figure 4:**
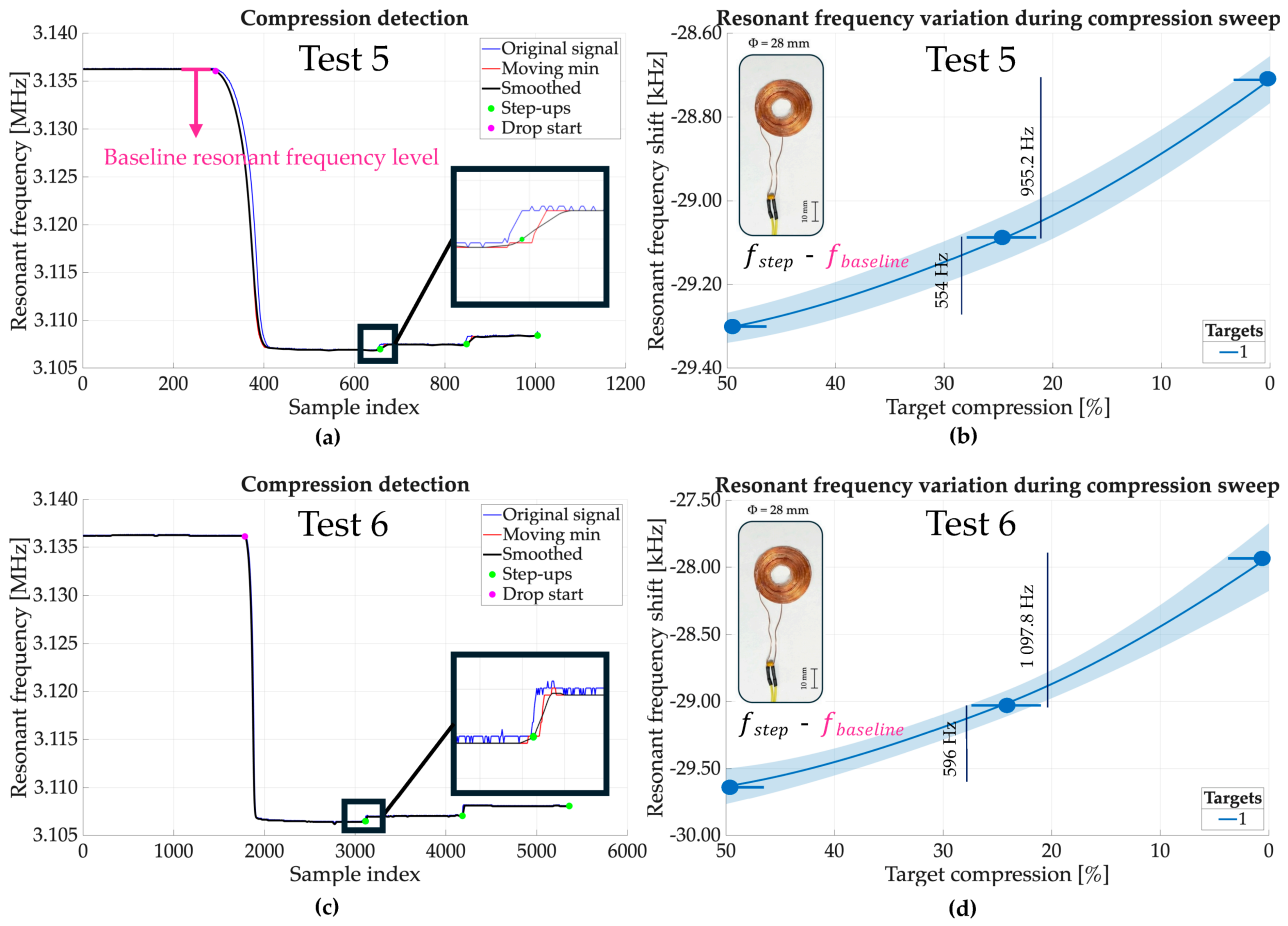
Resonant-frequency measurements of the medium LC resonator during controlled compression sweeps (50–25–0% compression strain). Short-duration compression test: (**a**) Measured resonant-frequency signal for the 1.00 mm thick target, showing the drop followed by step-up compression, together with the smoothed trace, moving-minimum estimate, and automatically detected transition points. (**b**) Corresponding resonant-frequency shift as a function of target compression, computed relative to the baseline resonant frequency. Shaded regions represent the standard deviation of repeated measurements. Longer duration compression test: (**c**) Measured resonant-frequency signal for the 1.00 mm thick target under long-duration tests, including the drop behavior, subsequent step-up sequence, smoothed signal, and detected transition points. (**d**) Resonant-frequency shift versus compression for the long-duration test, referenced to the pre-compression baseline and including the standard deviation of the measurements. For all right-hand panels, the continuous curves were obtained by cubic-spline interpolation of the discrete experimental measurements.

During the compression tests, the 1.00 mm thick target was subjected to controlled deformation, and the corresponding resonant frequencies were recorded, as shown in Figure 4a. A 50% compression produced a resonant-frequency shift of −29.28 kHz relative to the baseline condition (sensor positioned at a distance of 10 mm from the target). Reducing the compression from 50% to 25% increased the resonant frequency shift to −28.73 kHz, corresponding to a difference of 0.55 kHz (below 2SD(d)) between the two levels. Further reducing the compression from 25% to 0% increased the resonant-frequency shift to −27.78 kHz, yielding an additional difference of approximately 0.95 kHz, as shown in Figure 4b. Across repeated measurements, the average standard deviation (Mean SD) was 0.14 kHz, the standard uncertainty of the mean ($u_{A},\;$Type A) was 0.08 kHz and the 95% confidence interval (CI) was ±0.35 kHz.

The longer-duration compression test confirmed the same trend observed in the short-duration measurements, as illustrated in Figure 4c. The mean resonant-frequency shifts relative to the baseline were −29.63 kHz at 50% compression, −29.03 kHz at 25%, and −27.93 kHz at 0% compression. Accordingly, reducing compression from 50% to 25% produced a frequency increase of 0.60 kHz, while the transition from 25% to 0% yielded a further increase of approximately 1.10 kHz (Figure 4d). The average standard deviation (Mean SD) across repeated acquisitions was 0.24 kHz, the standard uncertainty of the mean ($u_{A},\;$Type A) was 0.14 kHz and the 95% confidence interval (CI) was ±0.59 kHz.

### 3.3. Temperature and Humidity Stability Tests

**Figure 5:**
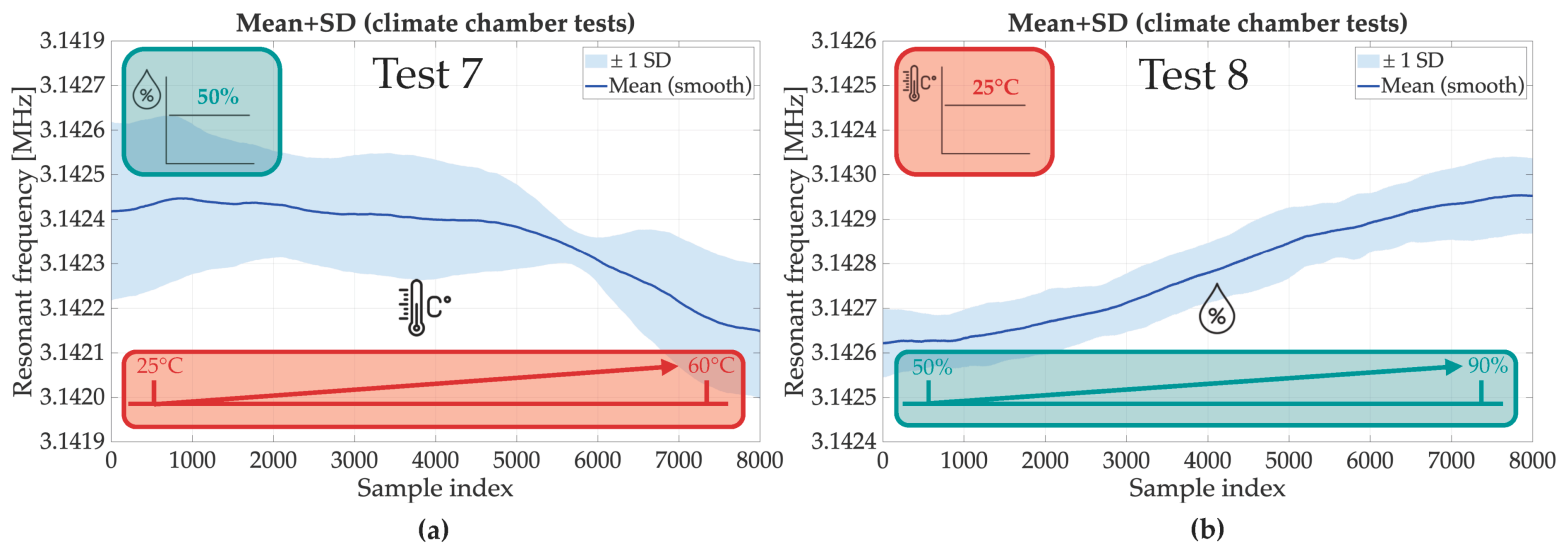
Resonant-frequency stability of the medium LC resonator under controlled environmental variations in the climate chamber. (**a**) Mean resonant-frequency trend and corresponding standard deviation during a temperature sweep from 25 °C to 60 °C, performed at a constant relative humidity (50%). (**b**) Mean resonant-frequency trend and corresponding standard deviation during a humidity sweep from 50% to 90%, performed at a constant temperature (25 °C).

For the temperature and humidity stability tests, the medium LC resonator paired with the 1.00 mm thick target was used. Figure 5a reports the mean resonant frequency and corresponding standard deviation computed over three repeated measurements, with the target and coil positioned at a fixed separation of 0 mm, relative humidity maintained at 50%, and temperature varied from 25 °C to 60 °C. Each measurement sequence consisted of 8000 samples. A decrease of approximately 0.30 kHz in the resonant frequency was observed relative to the initial condition. The average standard deviation (Mean SD) across the entire acquisition was 0.12 kHz and the standard uncertainty of the mean ($u_{A},\;$Type A) was 0.07 kHz. Under standard environmental conditions (25 °C and 50% relative humidity), the resonant frequency was 3.1424 MHz. When the temperature was increased to 60 °C at the same humidity level (50%), the measured resonant frequency was 3.1421 MHz. During the second set of tests, the temperature was kept constant at 25 °C while the relative humidity increased from 50% to 90%, with the target and coil maintained at a fixed separation of 0 mm. The results, shown in Figure 5b, indicate a slight increase in the resonant frequency, rising from 3.1425 MHz at 50% relative humidity to 3.1429 MHz at 90% relative humidity. Each measurement sequence consisted of 8000 samples and was repeated three times. Overall, the resonant frequency increased by approximately 0.40 kHz, with an average standard deviation (Mean SD) of 0.07 kHz and a standard uncertainty of the mean ($u_{A},\;$Type A) of 0.04 kHz.

### 3.4. In Vitro Validation

**Figure 6:**
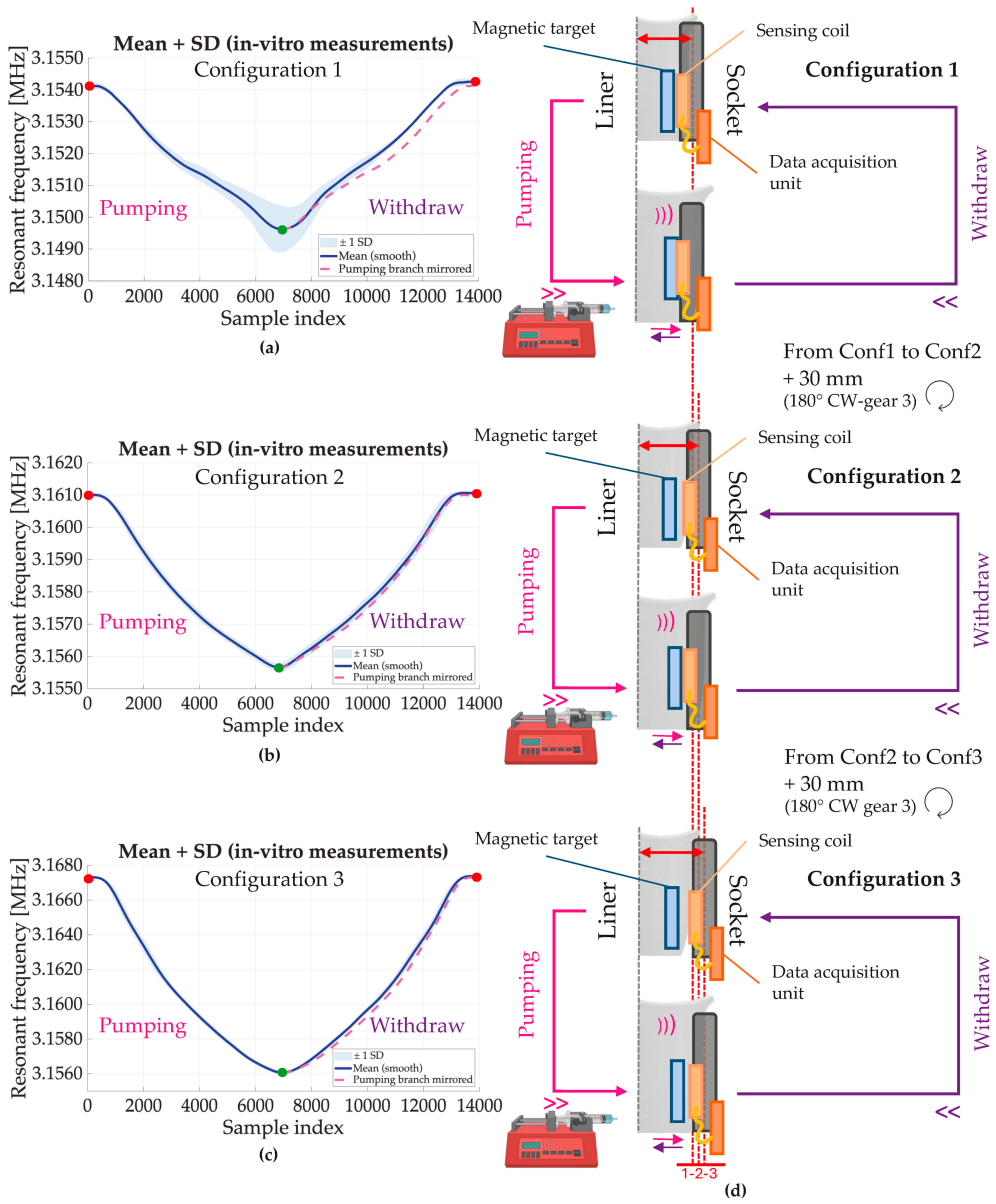
In vitro validation of the inductive sensing system using a transfemoral prosthetic socket and residual limb simulator under three different socket configurations. (**a**) Mean resonant-frequency trend (±standard deviation) recorded during three repeated inflation–deflation cycles for Configuration 1, corresponding to the nominal socket-fitting condition. The simulator volume was progressively increased by injecting water (pumping phase) up to 300 mL (+7.5% volume increment) and subsequently decreased through fluid withdrawal, returning to the initial state. (**b**) Mean resonant-frequency trend (±standard deviation) obtained for Configuration 2, achieved by untightening the BOA^®^ adjustment mechanism of 30 mm, thereby increasing the socket internal volume and modifying the liner–socket distance. The same controlled pumping and withdrawal procedure was applied. (**c**) Mean resonant-frequency trend (±standard deviation) measured for Configuration 3, achieved by untightening the BOA^®^ adjustment mechanism of 60 mm, further increasing the socket width. The resonant-frequency variation during the pumping and withdrawal phases reflects the cyclic changes in the liner–socket distance induced by the simulated residual limb volume variations. (**d**) Schematic representation of the experimental setup. A magnetic target is bonded to the external surface of the silicone liner worn on the residual limb simulator, while the sensing coil is embedded in the inner wall of the prosthetic socket and connected to the external data acquisition unit. Controlled volume variations are generated by syringe pumps through fluid injection and withdrawal. Pink arrows indicate the pumping direction, corresponding to fluid injection and expansion of the liner toward the socket, whereas purple arrows indicate fluid withdrawal and liner deflation. The diagram also illustrates the progressive socket adjustments between configurations obtained by rotating the BOA^®^ mechanism. A video demonstrating the experimental setup and measurement procedure is provided in Video S1.

Figure 6 illustrates the experimental setup and the resonant-frequency measurements obtained during the inflation–deflation cycles for the three tested socket configurations. In Configuration 1, corresponding to the nominal fitting condition with full liner–socket adherence, the mean resonant frequency measured at the initial non-inflated state was 3.154 MHz (differences in the absolute resonant frequency compared with the previous tests are attributed to changes in the surrounding environment and boundary conditions introduced by the in vitro setup, resulting in a different effective magnetic permeability around the sensor–target system). During the inflation phase, the resonant frequency progressively decreased as the simulator volume increased, reaching a minimum value of approximately 3.149 MHz at maximum inflation (300 mL, i.e., +7.5%). During the subsequent deflation phase, the resonant frequency increased again, returning to approximately 3.154 MHz once the simulator returned to its initial volume, as shown in Figure 6a. Across the repeated cycles, the measurements showed an average standard deviation (Mean SD) of 0.23 kHz, a standard uncertainty of the mean ($u_{A},\;$Type A) of 0.13 kHz and a 95% confidence interval (CI) of ± 0.58 kHz indicating stable and repeatable signal behavior. The average hysteresis between the inflation and deflation branches was 0.24 kHz, with a standard deviation of 0.17 kHz.

In Configuration 2, obtained by untightening the socket adjustment BOA^®^ mechanism by 30 mm, the baseline resonant frequency measured at the non-inflated condition increased to 3.161 MHz. As in the previous configuration, the resonant frequency decreased during the inflation phase, reaching approximately 3.156 MHz at maximum volume expansion. Following fluid withdrawal, the frequency increased again and returned close to the baseline value of 3.161 MHz, as shown in Figure 6b. The measurements exhibited an average standard deviation (Mean SD) of 0.16 kHz, a standard uncertainty of the mean ($u_{A},\;$Type A) of 0.09 kHz, and a 95% confidence interval (CI) of ±0.39 kHz, confirming the good repeatability of the sensing response. The average hysteresis between the inflation and deflation branches was 0.09 kHz, with a standard deviation of 0.05 kHz.

In Configuration 3, obtained by untightening the socket adjustment BOA^®^ mechanism by 60 mm, the baseline resonant frequency at the non-inflated condition further increased to 3.169 MHz. During the inflation phase, the resonant frequency decreased to approximately 3.158 MHz at maximum injected volume. After fluid withdrawal and restoration of the initial simulator volume, the resonant frequency returned to approximately 3.169 MHz, as shown in Figure 6c. The average standard deviation (Mean SD) measured across the repeated cycles was 0.09 kHz, the standard uncertainty of the mean ($u_{A},\;$Type A) was 0.05 kHz, and the 95% confidence interval (CI) was ±0.49 kHz. The average hysteresis between the inflation and deflation branches was 0.24 kHz, with a standard deviation of 0.15 kHz. Overall, across all three socket configurations, the sensing system consistently detected the cyclic variations induced by controlled volume changes of the residual limb simulator. The resonant frequency decreased during the inflation phase and increased during deflation, with repeatable responses across the repeated cycles and low measurement variability ($u_{A},\;$Type A < 0.13 kHz). These results confirm the capability of the inductive sensing system to detect socket–liner distance variations associated with residual limb volume fluctuations under different fitting conditions.

## 4. Discussion

The primary aim of this study was to develop, experimentally characterize and select an optimized configuration for an inductive sensing system designed to measure prosthetic socket fit. The inductive sensing system is a three-component system constituted by a portable data acquisition unit, an LC resonator and a magnetic silicone target.

Five different magnetic silicone targets were produced and evaluated using both industrial and custom-made LC resonators. Among the tested configurations, the 1.00 mm thick target with 70 wt% NdFeB particles provided the best performance, producing the largest resonant-frequency shift (29.84 kHz) together with a relatively low Type A standard uncertainty ($u_{A}$) of 0.21 kHz. Furthermore, the local sensitivity was 9.74 kHz/mm at near-contact conditions and 0.88 kHz/mm at the near-detection limit, corresponding to estimated local distance resolutions of 0.05 mm and 0.56 mm, respectively. The improved performance of the 1 mm thick magnetic target with 70 wt% NdFeB particles can be attributed to its greater magnetic volume, which enhances magnetic coupling with the sensing coil and induces larger inductance variations over distance while preserving sufficient mechanical compliance. Although particle loads between 75 wt% and 80 wt% have been reported in the literature (Henrikson et al. use 80 wt% incorporated into a proprietary polymer matrix [27]); our incorporation was limited to 70 wt% [26,31,32]. Indeed, exceeding 70 wt% would compromise the casting process of the Ecoflex™ 00-50 matrix due to excessively high viscosity and would make the final target more fragile and stiffer. Since the pressures at the socket–limb interface in transfemoral prostheses are extremely high, reaching peaks of 113.80 kPa during walking [11], the use of excessively loaded and stiff silicone targets would increase the risk of cracks and fractures, thereby reducing the durability of the system.

The final selection of the LC resonator was made by evaluating the stability and the noise of the measured signal. Among these, the medium LC resonator exhibited the most stable response and the lowest noise level, while also presenting the highest quality factor (Q = 21.7) compared with the industrial coil (Q = 4.5) and the large coil (Q = 17.0). In addition, the medium coil enabled the largest measurable displacement range (0–7.00 mm) and produced the largest overall frequency shift (29.84 kHz), confirming this configuration as the most suitable sensing solution for the intended application.

Following the selection of the reference configuration, additional testing was performed. Longer-duration distance tests performed with a robotic arm confirmed the stability of the sensor response over the 0–10 mm measurement range, with a Type A standard uncertainty ($u_{A}$) of 0.03 kHz, demonstrating reliable operation during prolonged acquisition periods. Compression experiments further showed that deformation of the magnetic silicone target produced measurable resonant-frequency variations, with frequency differences of approximately 0.60 kHz between 50% and 25% compression and 1.10 kHz between 25% and 0% compression (with a standard uncertainty of the mean ($u_{A}$), Type A, below 0.14 kHz), confirming the ability of the sensor to detect mechanical deformation of the liner material.

When considering the integration of the system inside a prosthetic socket, several practical aspects must be taken into account, including mechanical robustness and exposure to environmental factors such as sweat, humidity, and temperature variations. In this work, the influence of temperature and humidity was experimentally evaluated using a climatic chamber, and the results confirm that the system exhibits only minor resonant-frequency shifts under the tested conditions. Specifically, increasing the temperature from 25 °C to 60 °C produced a resonant-frequency change of approximately 0.30 kHz, while increasing the relative humidity from 50% to 90% resulted in a variation of about 0.40 kHz, values that remain negligible compared with the system measurement range and sensitivity.

Finally, the sensing system was validated in a high-fidelity transfemoral residual limb simulator combined with an adjustable prosthetic socket reproducing representative socket-fit conditions under controlled laboratory settings. Controlled limb volume variations of 300 mL (±7.5%) were generated through cyclic fluid injection and withdrawal. A unique conversion from the global volume change (+7.5%, 300 mL) to a local coil–target displacement cannot be established. The injected volume is distributed non-uniformly across the compliant limb surface and depends on socket geometry, liner deformation, and local contact conditions. Across three socket configurations, the system consistently detected repeatable resonant-frequency changes within an approximate range of 3.15–3.17 MHz, corresponding to frequency variations of up to 20 kHz, with a Type A standard uncertainty ($u_{A}$) below 0.13 kHz. These results confirm the capability of the proposed inductive sensing system to reliably detect socket–liner distance variations associated with residual limb volume fluctuations. The observed hysteresis is likely associated with the viscoelastic behavior of the compliant silicone-based materials used in the experimental setup, including the magnetic target, liner, and residual limb simulator. Their time-dependent mechanical behavior during cyclic loading may contribute to the small differences observed between the loading and unloading branches.

It should be noted that, in the prosthetic socket configuration, the measured resonant-frequency shift reflects the combined effects of changes in coil–target distance and compression of the compliant magnetic target arising from interface loading. Compression reduces the target thickness and may, therefore, be interpreted as a reduction in the effective electromagnetic separation between the sensing coil and the magnetic material. Consequently, both mechanisms produce resonant-frequency shifts with the same sign and cannot be uniquely separated using the current single-channel sensing configuration. To quantify their relative influence, separate characterization experiments were performed. Under the investigated operating conditions, variations in the coil–target distance of 1.00 mm produced a resonant-frequency shift of approximately 10 kHz, whereas even a severe target compression of up to 50% (an approximately 0.5 mm reduction in target thickness) produced a shift of only 1–1.5 kHz. Therefore, although target compression contributes to the measured signal, its effect is approximately one order of magnitude smaller than that associated with coil–target distance variation over the investigated operating range. For the intended clinical application, however, the most clinically relevant output is not the isolated estimation of coil–target distance or target compression but rather an overall indicator of the socket-fit condition. This interpretation is consistent with previous studies employing inductive sensing for socket-fit monitoring, where the measured signal is interpreted as a clinically meaningful indicator of the socket-fit condition rather than as a direct measurement of a single mechanical variable [33]. Future work will investigate a patient-specific calibration procedure and a two-regime signal-interpretation strategy. During the gap-closing regime, the sensor response could primarily be used to detect changes in coil–target separation and identify the onset of contact. Once contact has been established, lower-magnitude frequency variations could be interpreted using a target–compression calibration curve to monitor changes in the post-contact mechanical state of the socket–liner interface. Rather than using the compression-related response during continuous gait monitoring, this assessment could be performed under standardized static conditions, for example while the user is standing still, and could be voluntarily activated at selected times, such as after donning the prosthesis, during periodic socket-fit checks, or when discomfort is perceived. Based on the present characterization results, it is anticipated that, during dynamic activities such as walking, the additional contribution of target compression will remain smaller than the variations associated with changes in coil–target separation. Consequently, continuous monitoring during gait would primarily reflect changes in coil–target distance, whereas the proposed calibration procedure would be reserved for standardized static assessments of the post-contact mechanical state of the socket–liner interface. This user-initiated approach could reduce the influence of dynamic loading, motion-related artifacts, and transient misalignment, while still providing clinically useful information when socket adjustment may be required. The feasibility and robustness of this strategy will be investigated and validated in future studies involving prosthesis users under different weight-bearing, alignment, and interface conditions. In addition, future studies will assess the intermediate reproducibility of the proposed sensing system, including sensor re-mounting, target re-fabrication, and measurements performed on different days, as well as the influence of coil–target misalignment under representative prosthetic-use conditions.

To better contextualize the proposed sensing approach within the current state of the art, Table 7 compares its main characteristics with those of the principal sensing technologies adopted for prosthetic socket monitoring. This study confirms the feasibility of the proposed inductive technology for the target application, offering a robust and compact alternative that overcomes the drift and accuracy issues associated with conventional pressure sensors such as FSRs. A systematic characterization and optimization of the electromagnetic components is presented, including a comparison of multiple coil geometries and target compositions, which enabled the identification of a configuration (medium coil and 1 mm thick target) capable of extending the detection range up to 7.00 mm. This represents a critical improvement for application in a transfemoral prosthetic socket, as transfemoral residual limb volume fluctuations can be highly pronounced [10]. Furthermore, unlike systems based on “proximity counts” (frequency ratios) [27], our system implements direct monitoring of the resonant frequency. This technical choice offers a more linear and physically interpretable measure of electromagnetic interaction, facilitating calibration and improving signal stability.

In future work, the performance of the LC resonator could be further enhanced by adopting a multilayer configuration for the sensing coil. This approach would allow the number of turns and the inductance to be increased within a compact diameter, thereby maximizing the measurable frequency variation. Further improvements may also include the integration of a ferrite sheet behind the coil to concentrate the magnetic flux density and the use of stranded enameled copper wire (Litz wire) to reduce skin-effect losses [34]. In addition, the LC circuit will be printed or integrated on a PCB on which SMD capacitors can be soldered, instead of using self-built or industrial sensors, in order to further compact and stabilize the sensing system.

## Figures and Tables

**Table 1:** Comparison of the sensing-coil characteristics (**top**) and corresponding LC resonator circuit parameters (**bottom**). The resonant frequency values were obtained from VNA measurements and are reported as the mean ± standard deviation (N = 3). The electrical resistance was measured using an LCR meter and is likewise reported as the mean ± standard deviation (N = 3). The inductance values were not directly measured; they were calculated from the measured resonant frequency and the nominal capacitance connected in parallel to each coil. Capacitor values refer to their nominal specifications.

**Sensing Coils**	**Industrial**	**Medium**	**Large**
Wire diameter (mm)	0.30	0.30	0.30
Inner diameter (mm)	12	10	10
Outer diameter (mm)	20	28	34
Turns	10	25	30
Layers	2	1	1
**LC resonators**	**Industrial**	**Medium**	**Large**
Resonant frequency (MHz)	0.98 ± 0.01	3.32 ± 0.02	3.38 ± 0.02
Quality factor	4.50	21.70	17.0
Capacitance (pF)	2200	181	101
Inductance (μH)	12.00	12.70	21.8
Resistance (ohm)	0.40 ± 0.05	0.50 ± 0.05	0.70 ± 0.05

**Table 2:** Targets: magnetized silicone particle contents and thicknesses.

Particle Content (wt%)	Measured Thickness (mm)	Target Thickness (mm)
70	0.44 ± 0.02	0.50
70	0.65 ± 0.02	0.50
70	1.00 ± 0.01	1.00
60	0.38 ± 0.02	0.50
60	0.72 ± 0.01	1.00

**Table 3:** Summary of the LC resonator-target combinations tested during the distance–frequency experiments. The large resonator was evaluated only with the 1 mm target, as the other target thicknesses had already shown suboptimal performance during the industrial and medium coil trials. Preliminary short-duration tests demonstrated that the medium coil combined with the 1 mm target provided the most stable and low-noise response; therefore, this optimal configuration was selected for long-duration measurements.

LC Resonator	Magnetic Targets	Duration	Type	Test Index
Industrial	All	Short duration	Distance 0–10	1
Medium	All	Short duration	Distance 0–10	2
Large	1 mm thickness	Short duration	Distance 0–10	3
Medium	1 mm thickness	Long duration	Distance 0–10	4

**Table 4:** Summary of resonant-frequency variations during the distance sweep relative to the baseline condition (10 mm sensor–target separation), measurement uncertainty, and detectable distance range for the magnetic targets evaluated with the industrial LC resonator. Resonant-frequency shifts are reported as Δ*f* = *f_measured_* − *f_baseline_*, where *f_baseline_* is the resonant frequency measured at a sensor–target separation of 10 mm. Measurement uncertainty is reported as the Type A standard uncertainty of the mean ($u_{A}=SD/\sqrt{N}$, k=1), calculated from three repeated measurements (N = 3). For completeness, the corresponding two-sided 95% confidence interval was calculated as $CI95=t_{0.975,2}u_{A}$. The detectable distance range was defined as the largest continuous interval satisfying |Δf(d)|≥2 SD(d), where SD(d) is the local standard deviation at each distance. Local sensitivity was calculated using the finite difference between consecutive measurement points $S(d_{i}\;)=\;({∆f}_{i+1}-{∆f}_{i})/(d_{i+1}-d_{i}),$ while the distance resolution was estimated to be ∂d= 2 SD(d)/S(d).

	Target				
	1.00 mm	0.72 mm	0.65 mm	0.44 mm	0.38 mm
Δf (at 0 mm) and Δf (at detection limit)	from −13.08 to −2.93 kHz	from −10.91 to −4.33 kHz	from −10.76 to −6.04 kHz	from −10.14 to −4.55 kHz	−2.66 kHz
Total Δf shift range	13.07 kHz	11.92 kHz	10.87 kHz	9.51 kHz	3.92 kHz
Mean SD	1.02 kHz	1.20 kHz	0.99 kHz	1.45 kHz	2.43 kHz
u_A_ (Type A)	0.59 kHz	0.69 kHz	0.57 kHz	0.84 kHz	1.40 kHz
95% CI (kHz)	±2.52 kHz	±2.96 kHz	±2.46 kHz	±3.61 kHz	±6.03 kHz
Detectable range	0–2.00 mm	0–1.00 mm	0–1.00 mm	0–1.00 mm	<1.00 mm
Near contact local sensitivity	6.09 kHz/mm	6.58 kHz/mm	4.73 kHz/mm	5.59 kHz/mm	2.38 kHz/mm
Distance resolution	0.39 mm	0.26 mm	0.87 mm	0.65 mm	3.57 mm
Near detection limit local sensitivity	4.06 kHz/mm	2.41 kHz/mm	3.48 kHz/mm	2.71 kHz/mm	0.17 kHz/mm
Distance resolution	0.68 mm	0.83 mm	0.10 mm	1.40 mm	48.67 mm

**Table 5:** Summary of resonant-frequency variations during the distance sweep relative to the baseline condition (10 mm sensor–target separation), measurement uncertainty, and detectable distance range for the magnetic targets evaluated with the medium LC resonator. Resonant-frequency shifts are reported as *Δf = f_measured_ − f_baseline_*, where *f_baseline_* is the resonant frequency measured at a sensor–target separation of 10 mm. Measurement uncertainty is reported as the Type A standard uncertainty of the mean ($u_{A}=SD/\sqrt{N}$, k=1), calculated from three repeated measurements (N = 3), where SD is the sample standard deviation of the repeated measurements. For completeness, the corresponding two-sided 95% confidence interval was calculated as $CI95=t_{0.975,2}u_{A}$. The detectable distance range was defined as the largest continuous interval satisfying |Δf(d)|≥2 SD(d), where SD(d) is the local standard deviation at each distance. Local sensitivity was calculated using the finite difference between consecutive measurement points $S(d_{i})=\;({∆f}_{i+1}-{∆f}_{i})/(d_{i+1}-d_{i}),$ while the distance resolution was estimated as ∂d= 2 SD(d)/S(d).

	Target				
	1.00 mm	0.72 mm	0.65 mm	0.44 mm	0.38 mm
Δf (at 0 mm) and Δf (at detection limit)	from −31.37 to −2.94 kHz	from −23.11 to −2.52 kHz	from −25.82 to −2.63 kHz	from −21.67 to −2.52 kHz	from −17.24 to −2.39 kHz
Total Δf shift range	29.84 kHz	21.57 kHz	25.58 kHz	20.40 kHz	14.85 kHz
Mean SD	0.36 kHz	0.91 kHz	0.74 kHz	0.16 kHz	0.21 kHz
u_A_ (Type A)	0.25 kHz	0.54 kHz	0.42 kHz	0.09 kHz	0.12 kHz
95% CI	±1.07 kHz	±2.33 kHz	±1.81 kHz	±0.69 kHz	±0.65 kHz
Detectable range	0–7.00 mm	0–6.00 mm	0–6.0 mm	0–6.0 mm	0–6.0 mm
Near contact local sensitivity	9.74 kHz/mm	7.60 kHz/mm	8.52 kHz/mm	7.21 kHz/mm	5.71 kHz/mm
Distance resolution	0.05 mm	0.26 mm	0.22 mm	0.04 mm	0.08 mm
Near detection limit local sensitivity	0.88 kHz/mm	0.91 kHz/mm	0.99 kHz/mm	0.88 kHz/mm	0.62 kHz/mm
Distance resolution	0.56 mm	1.68 mm	1.22 mm	0.40 mm	1.37 mm

**Table 6:** Comparison of the results obtained from measurements of the three LC resonators over a distance range of 0–10 mm for the 1 mm thick magnetic target.

LC Resonator Coil	Industrial	Medium	Large
Signal	Stable but noisy	Stable and less noisy	Unstable and noisy
Detectable range	0–2.00 mm	0–7.00 mm	0–4.00 mm
Total frequency shift	13.07 kHz	29.84 kHz	24.00 kHz
Mean SD	1.02 kHz	0.36 kHz	2.23 kHz
u_A_ (Type A)	0.59 kHz	0.21 kHz	1.29 kHz
95% CI	±2.52 kHz	±1.07 kHz	±5.55 kHz
Local sensitivity	4.06 kHz/mm	9.74 kHz/mm	11.10 kHz/mm
Distance resolution	0.39 mm	0.05 mm	0.47 mm

**Table 7:** Quantitative and qualitative performance comparison between the proposed inductive sensing approach and representative prosthetic socket sensing technologies [13,14].

Technology	Measured Quantity	Sensing Range	Reported Stability	Thickness	Electronic Complexity	Validation Setting
FSRs [14,15]	Interface pressure	300 kPa	High drift (5–30%)	0.20–0.48 mm	Low	Clinical (widely used)
Capacitive [13,21]	Pressure	0–350 kPa	Moderate error (4–24%)	0.63–4.00 mm	High	Experimental (small scale)
Fiber optic (FBG) [19,20]	Pressure	<200 kPa	Negligible drift	2.00–3.00 mm (pad)	High	Experimental (in situ)
Prev. inductive [26,27]	Distance	0–15 mm	Low thermal drift (3%)	0.15 mm (coil)	Moderate	Clinical (small scale)
Proposed system	Distance/fit (freq. shift)	0–7.00 mm	Low variability (1–2%)	1.00 mm (target)	Low	Laboratory (in vitro)

## Data Availability

The codes and datasets generated in this study are available from the corresponding author upon reasonable request. Public access is restricted due to privacy considerations and the ongoing nature of the research project.

## Supplementary Materials

The following supporting information can be downloaded at: https://zenodo.org/records/20718871 (accessed on 22 July 2026), Video S1: Demonstration of the experimental setup and data acquisition procedure used for the in vitro validation of the inductive sensing system, including the prosthetic socket configuration and the controlled pumping–withdrawal cycles of the residual limb simulator.

## Abbreviations

The following abbreviations are used in this manuscript:

FSRs	Force-Sensitive Resistors
LC	Inductance Capacitance
LDC	Inductance-to-Digital Converter
EVM	Evaluation Module
Q	Quality factor
